# Cell Type–Specific Transcriptome Analysis Reveals a Major Role for *Zeb1* and miR-200b in Mouse Inner Ear Morphogenesis

**DOI:** 10.1371/journal.pgen.1002309

**Published:** 2011-09-29

**Authors:** Ronna Hertzano, Ran Elkon, Kiyoto Kurima, Annie Morrisson, Siaw-Lin Chan, Michelle Sallin, Andrew Biedlingmaier, Douglas S. Darling, Andrew J. Griffith, David J. Eisenman, Scott E. Strome

**Affiliations:** 1 Department of Otorhinolaryngology–Head and Neck Surgery, University of Maryland, Baltimore, Maryland, United States of America; 2 Division of Gene Regulation, The Netherlands Cancer Institute, Amsterdam, The Netherlands; 3 Molecular Biology and Genetics Section, Otolaryngology Branch, National Institute on Deafness and Other Communication Disorders, National Institutes of Health, Rockville, Maryland, United States of America; 4 Department of Oral Health and Rehabilitation and Center for Genetics and Molecular Medicine, University of Louisville, Louisville, Kentucky, United States of America; The Jackson Laboratory, United States of America

## Abstract

Cellular heterogeneity hinders the extraction of functionally significant results and inference of regulatory networks from wide-scale expression profiles of complex mammalian organs. The mammalian inner ear consists of the auditory and vestibular systems that are each composed of hair cells, supporting cells, neurons, mesenchymal cells, other epithelial cells, and blood vessels. We developed a novel protocol to sort auditory and vestibular tissues of newborn mouse inner ears into their major cellular components. Transcriptome profiling of the sorted cells identified cell type–specific expression clusters. Computational analysis detected transcription factors and microRNAs that play key roles in determining cell identity in the inner ear. Specifically, our analysis revealed the role of the *Zeb1*/miR-200b pathway in establishing epithelial and mesenchymal identity in the inner ear. Furthermore, we detected a misregulation of the ZEB1 pathway in the inner ear of Twirler mice, which manifest, among other phenotypes, malformations of the auditory and vestibular labyrinth. The association of misregulation of the ZEB1/miR-200b pathway with auditory and vestibular defects in the Twirler mutant mice uncovers a novel mechanism underlying deafness and balance disorders. Our approach can be employed to decipher additional complex regulatory networks underlying other hearing and balance mouse mutants.

## Author Summary

The mammalian inner ear is a highly complex sensory organ, and mutations in more than 100 genes underlie hereditary human non-syndromic hearing loss. Nevertheless, little is known about the signaling cascades downstream of deafness genes. Genome-wide expression profiling is an invaluable tool for gaining systems-level understanding of biological processes. We developed and validated a simple and novel protocol to isolate sensory epithelial cells, neurons, blood vessels, and mesenchyme of auditory and vestibular epithelia from newborn wild-type mice. Our protocol is based on flow cytometry to sort and capture cells labeled with commercially available antibodies to endogenously expressed cluster of differentiation (CD) antigens. Using this strategy, we identified Zeb1 and miR200b as regulators of epithelial and mesenchymal identity in the mouse inner ear, and we further identified the signaling pathway disrupted by the *Zeb1* mutation in the Twirler mouse mutant. We also show the utility of this approach for characterizing compartment-specific genes and protein–protein networks. Implementation of this isolation strategy to study other mouse mutants with hearing and balance phenotypes could overcome many of the obstacles to understanding the function of deafness genes.

## Introduction

Genome-wide expression profiling is a valuable tool for gaining systems-level understanding of biological processes during development, response to stress, and pathological conditions. However, accurate interpretation of expression profiles from complex tissues such as neuroepithelia is often complicated and hindered by cellular heterogeneity. Such cellular complexity has made it particularly difficult to identify relevant transcriptional networks from the auditory and vestibular systems of mammalian inner ears, which are composed of hair cells, multiple types of supporting cells, neurons, mesenchymal cells and vascular endothelium.

Hereditary hearing loss (HHL) is a common congenital sensory disability, affecting 1 in 2000 newborns and a significant portion of the elderly population. The complexity of the auditory and vestibular systems is reflected in over 250 genes which, when mutated, underlie inner ear malformations or dysfunction in mice (http://hearingimpairment.jax.org/master_table.html). Furthermore, there are over 118 syndromes that include hearing loss as part of their phenotype [1], and over 100 genes – roughly half of which have been cloned which underlie hereditary non-syndromic hearing loss in human (http://hereditaryhearingloss.org/) and [2]. The human and mouse inner ears are remarkably similar and the mouse has proven to be an invaluable tool in the study of hearing loss [3]. Nevertheless, cell type–specific molecular differences between the auditory and vestibular systems, and the signaling cascades upstream and downstream of most of the deafness genes have not been fully deciphered.

In this study we demonstrate the utility of endogenously expressed cell surface markers for separating the auditory and vestibular tissues into their major cellular components. We used a cell type–specific transcriptome analysis to identify regulators of cell fate determination in the inner ear. Finally, utilizing the example of the ZEB1/miR-200b pathway, we present a proof-of-concept that cell type–specific gene expression profiles can be used to identify molecular pathways upstream and downstream of deafness genes.

## Results

### A novel cell type–specific protocol to sort the inner ear sensory organs

**Figure 1:**
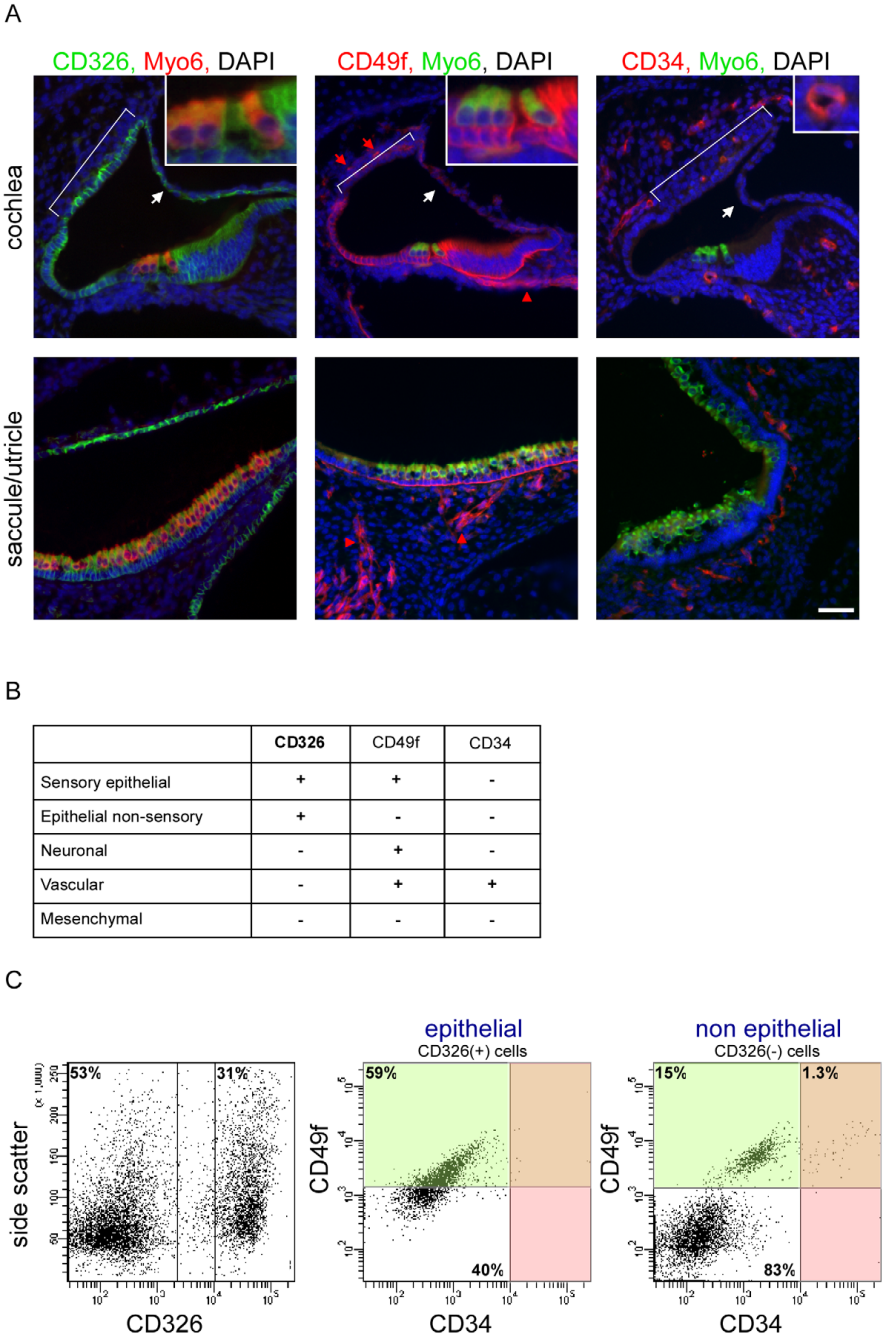
A novel cell type–specific protocol to sort the inner ear sensory organs. [A] Expression of CD326, CD49f and CD34 in the newborn mouse inner ear. Sections of P0 cochlear ducts (upper panel) and utricles/saccules (lower panels) from mouse inner ears immunolabeled with antibodies for CD326 (left panel), CD49f (middle panel) and CD34 (right panel), and counter-stained with an antibody for Myo6 – a hair cell-specific protein in the mouse inner ear, and DAPI (blue). CD326 labels all of the epithelial cells in the auditory (cochlea) and vestibular (saccule, utricle and semicircular canals) organs including the non-sensory epithelial cells of Reissner's membrane (white arrows) and the stria vascularis (bracket) in the cochlea. CD49f marks the sensory epithelium as well as the neuronal (red arrowhead) and vascular endothelial cells (red arrow). CD34 is specifically expressed in the vascular endothelium, thereby marking the blood vessels. Scale bar  =  50 µm, insets  =  150 µm. [B] Cell type–specific CD expression. [C] FACS plot analysis from newborn auditory epithelia of wild type mice. Cells are sorted based on expression of CD326 (here 53% and 31% negative and positive, respectively), are further divided based on the expression of CD49f and CD34 (for CD326-positive cells 59% and 40% are CD49f positive and negative, respectively; for CD326-negative cells 16.3% and 83% are CD49f positive and negative, respectively. 1.3% of the CD-326 negative cells are CD49f and CD34 positive). For simplification - the area marked in green represent CD49f-positive cells, and the area marked in red represent CD34-positive cells. See also Figure S1.

Our first goal was to develop a protocol for dissociating the inner ear sensory tissues into their major cellular components. We studied the ears of newborn mice to increase the likelihood of identifying genes that are important both for early and terminal differentiation of the inner ear. To identify antibodies that could be used to sort the inner ear into its major cellular compartments, we stained inner ears of P0 wild-type mice with commercially available monoclonal antibodies to the protein products of cell surface cluster of differentiation (CD) genes that are expressed in the ear [4]. We found that CD326 (EpCAM) is detected in all *sensory* and *non-sensory epithelial cells* of the mouse inner ear (Figure 1A). For the purpose of this manuscript, we define the cochlear sensory epithelium as the hair cells, supporting cells and cells of the greater and lesser epithelial ridges (i.e. epithelial cells that are not part of the stria vascularis or Reissner's membrane), and the vestibular sensory epithelium as hair cells and supporting cells. In contrast to CD326, the epithelial staining of CD49f (Integrin α6) is specific to the *sensory epithelial cells*. Within the non-epithelial cells, CD49f stains the neuronal and vascular endothelial cells. Finally, antibodies against CD34, a cell surface protein that is expressed on hematopoietic stem cells and vascular endothelium [5], specifically and uniquely stain the vascular endothelium in the inner ear. We postulated that inner ear cells can be divided into epithelial and non-epithelial cells based on the expression of CD326 and further divided into sensory epithelial, non-sensory epithelial, neuronal, vascular endothelial and mesenchymal cells based on the expression of CD49f and CD34 (Figure 1B).

Flow cytometric analysis of microdissected and dissociated tissues from the auditory and vestibular organs of postnatal day 0 to 1 (P0-P1) wild-type mice revealed a clear separation of vascular endothelial, mesenchymal, neuronal and epithelial cells. Sensory epithelial cells could be distinguished from non-sensory epithelial cells, forming a total of five cellular populations (Figure 1C, Figure S1A). Notably, the proportions of specific populations differed between the auditory and vestibular tissues. For example, the proportion of vascular endothelial cells is greater than three-fold higher in the vestibular tissues compared with the cochlea (p = 0.006, Figure S1B). As we could sort up to four cellular populations simultaneously, we decided to focus our experiments on a total of eight cellular populations – sensory epithelial, neuronal, vascular endothelial and mesenchymal cells from the auditory and vestibular organs.

To further test the purity of the sorted cells, total RNA was extracted from each of the eight sorted populations and semi-quantitative real time RT-PCR was performed using primers for the RNA transcripts encoding CD326, CD49f and CD34. All sorted cells showed 100- to 1000-fold enrichment for the mRNA that encode the markers used to sort their respective populations, supporting the molecular purity of the sorted populations (Figure S1C).

### Cell type–specific transcriptome analysis of the inner ear

**Figure 2:**
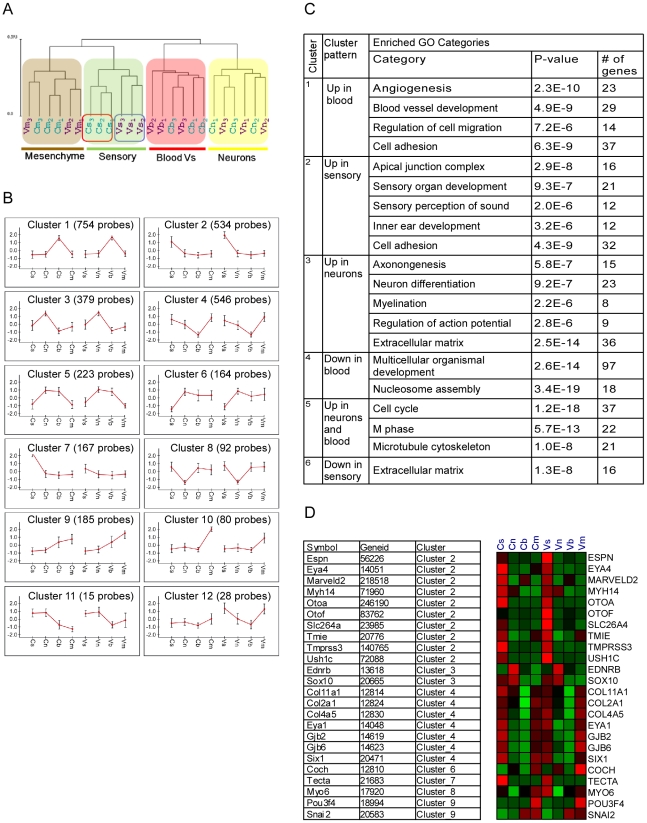
Analysis of the inner ear cell type–specific transcriptome. [A] Hierarchical clustering of the expression data resulted in a dendrogram in which the main partition of samples is according to cell-type (s-sensory, m-mesenchymal, n-neuron and b-blood cells). The auditory (C) and vestibular (V) samples clustered into separate branches only for the sensory epithelial cells. Samples from the auditory and vestibular systems are marked in blue and purple, respectively. Numbers represent the independent biological repeats. [B] Main expression patterns exhibited by the differential genes as identified by k-means clustering. Each cluster is represented by its mean expression pattern ± SD. (Prior to clustering, gene expression levels were standardized to mean = 0, SD = 1. Y-axis in the cluster view shows the standardized levels). At the top of each pattern, the title indicates the cluster number and the number of probes assigned to the cluster. Table S2 contains a list of the genes in each cluster. [C] A table depicting the observed enriched Gene-Ontology (GO) functional groups in six of the clusters. [D] A heat map depicting the expression patterns of deafness-related genes (right) and their assignment to the clusters (left). Red and green indicate increased and decreased expression, respectively.

We obtained wide-scale expression profiles of total RNA extracted from sensory epithelial, neuronal, mesenchymal and vascular endothelial cells of the auditory and vestibular tissues of newborn wild-type mice (a total of eight populations). We used the Illumina mouse expression arrays with probes for the majority of RefSeq-annotated genes (>24,000 genes), and over 7,000 predicted genes to generate eight ‘cell-type specific’ transcriptomes. We generated biologically independent triplicates for each cellular population, yielding a dataset of 24 independent transcriptomes. In the first step of the analysis of this dataset, we examined the overall similarity relations between the 24 transcriptome profiles. To accomplish this goal we subjected the profiles to hierarchical clustering which orders them in the structure of a hierarchical tree (dendrogram) in which similar transcriptomes are close to each other, while disparate ones are far apart in the tree. Our dataset's dendrogram clearly contains four main branches corresponding to the four cell types from which expression profiles were obtained (Figure 2A). This structure indicates that the characteristics of the transcriptomes primarily correspond to cell type rather than tissue from which they were obtained. Interestingly, sensory epithelial cells, but not other cell types from the auditory and vestibular tissues, were distinguished by sub-branches. This is consistent with the differentiated and specific functional characteristics of the sensory epithelium in auditory versus vestibular organs.

We next examined the dataset for genes whose expression varied significantly among the eight sorted cell populations. Two-way ANOVA tests [6] detected more than 3,000 differentially expressed genes (p<10^−5^, FDR<1%, Table S1). We then applied a clustering analysis which distributed these differentially expressed genes into sets based upon distinctive expression patterns [7]. Consistent with the hierarchical clustering results, the major clusters which were found in our dataset showed symmetric patterns of expression between the auditory and vestibular systems in the vast majority of clusters (Figure 2B and Table S2). For example, the largest clusters contained genes that were highly expressed in the vascular endothelium (cluster #1, 754 genes) or sensory epithelial cells (cluster #2, 534 genes), in both the auditory and vestibular organs. In contrast, cluster #7 (126 genes) contained genes that were highly expressed only in the auditory sensory cells but not in the corresponding cell population in the vestibular system.

To correlate the observed cellular transcriptomes with biological functions, we searched for enrichment of Gene-Ontology (GO) functional groups in each of the clusters [8]. All cell type–specific clusters were significantly enriched for functional categories that corresponded with the known roles of the cells (Figure 2D). For example, the clusters that contained genes highly expressed in vascular endothelial, sensory or neuronal cells were significantly enriched for genes which function in ‘angiogenesis’, ‘sensory perception of sound’ or ‘neuron differentiation’, respectively, further validating the accuracy of our sorting approach.

### Expression patterns of deafness-related genes

If differential gene expression implies functional significance, then deafness genes should be over represented in the clusters of differentially expressed genes. Forty-four of 66 genes known to underlie hereditary hearing loss (syndromic or non-syndromic –Table S3) were detected as expressed in our dataset. These genes were significantly over-represented 2.5-fold in the set of genes differentially expressed between the cell populations studied. Twenty-four (55%) of the deafness genes were differentially expressed in our dataset (compared with 22% predicted by random distribution, p = 1.2*10^−6^). Furthermore, the distribution of the deafness-related genes was significantly biased towards clusters 2 and 4 which contain genes highly expressed in sensory epithelial or sensory epithelial and mesenchymal cells, respectively (p = 0.00025, hyper-geometric tail, Figure 2D).

We reasoned that additional, yet undiscovered, deafness-related genes may have cell type–specific expression patterns that could be detected using our dataset. As a proof of principle, we searched our dataset of gene expression for positional candidate genes in Auditory Neuropathy Locus 1 (AUNA1) [9]. Prior to being cloned, this deafness locus spanned 18.3 megabases and contained 47 protein coding genes. Based on our database, 26 of the protein-coding genes were expressed in the ear, however, only two of these genes were selectively expressed in the neuronal cells of the auditory and vestibular systems: *Diap3*, whose human ortholog is now known to underlie AUNA1, and *Trim13* (Figure S2). Hence a cell type–specific expression analysis of AUNA1 could have prioritized two genes for analysis.

### Inner ear cell type–specific expression profiles identify candidate genes for deafness

**Figure 3:**
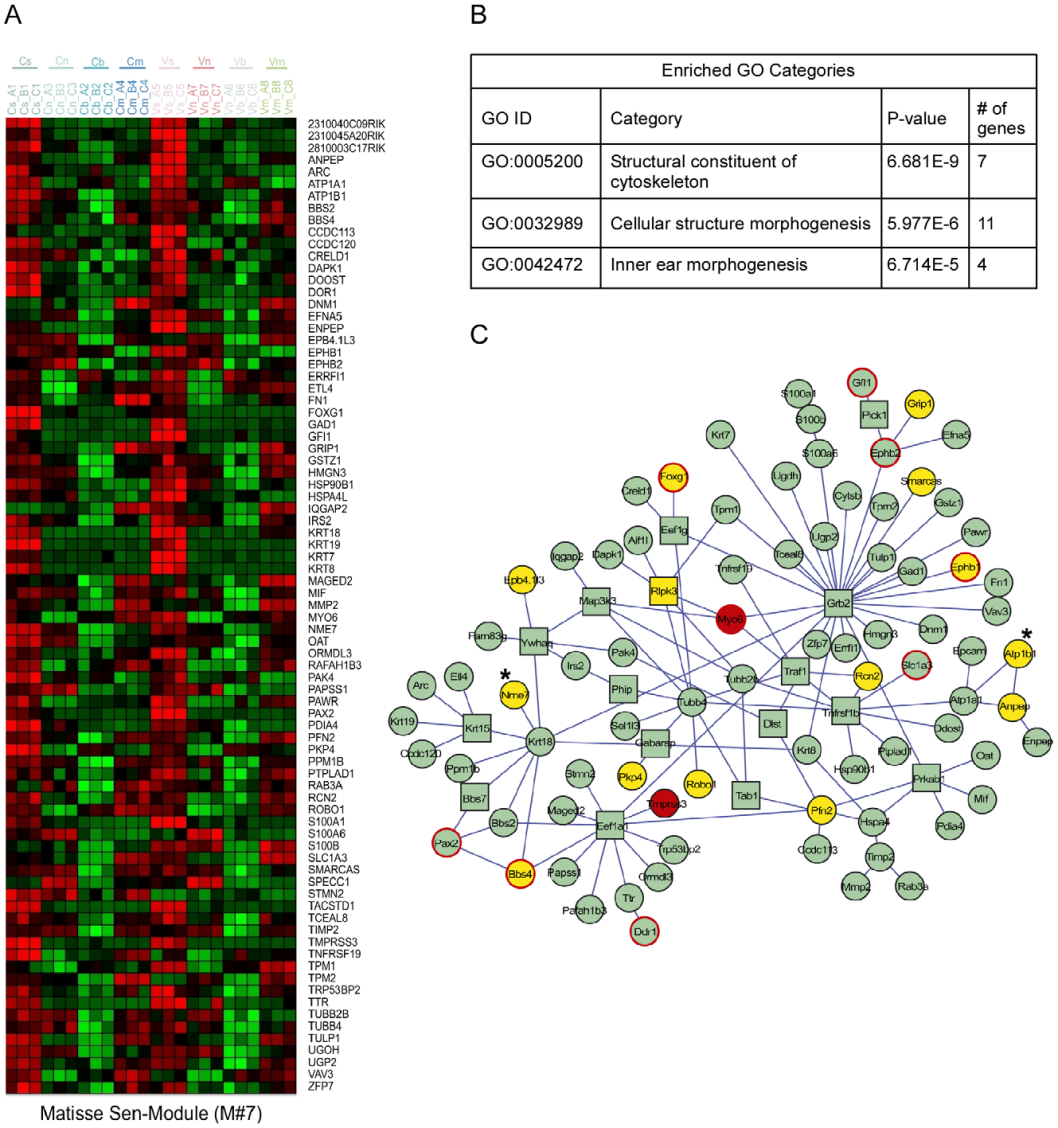
*Expression-interaction module* of genes which are highly expressed in sensory cells of the inner ear and are physically connected in the cellular protein-protein interaction web. [A] Heat map showing the inner ear expression pattern of the genes in this module. [B] GO functional categories which are statistically over-represented in this module. [C] Physical links between the proteins encoded by the genes of this module. Node's shape (circle verses square): several proteins were added to the module by the algorithm to keep other members connected although they do not share the module's characteristic expression pattern. These proteins are displayed by box nodes. Node's color: Known deafness-related genes are marked in red; genes which are located within deafness loci are marked in yellow. Genes whose mutations were reported to result in malformation of the inner ear in mouse but do not underlie human disease are marked by red frame. *ATP1B1* and *NME7* are candidate genes for *DFNA7* and are marked with asterisks. See also Figure S3.

As functionally related proteins often physically interact, we conducted an integrated analysis to search for groups of genes that both show similar expression patterns in the inner ear and are physically linked in the cellular web of protein-protein interactions [10]. Several *expression-interaction modules* were identified in our dataset (Figure 3 and Figure S3). Significantly, Module #7 contained genes highly expressed in sensory cells and was enriched for proteins that function in ‘inner ear morphogenesis’ (Figure 3B). This physically connected network contains two known non-syndromic deafness genes *MYO6* (MIM ID 600970) and *TMPRSS3* (MIM ID 605511). We speculated that defects in other proteins in this module could underlie hereditary hearing loss. To identify novel candidates, we correlated the genomic location of the human orthologues of the genes encoding the proteins in this expression-interaction module with the genomic intervals of the deafness loci for which the disease-causing genes are not yet identified. We found that the human orthologues of 15 of the genes in module #7 map to linkage intervals of uncloned deafness loci (Figure 3C). For example, *ATP1B1* and *NME7* are candidate genes for *DFNA7* based both on enrichment in the sensory epithelial cells and their involvement in this protein-protein interaction sub-network (Figure 3C, marked with asterisks). For ten of the deafness loci, our analysis identified candidate genes that are connected to known deafness genes by the protein-protein interaction network (Table S4). With the increased use of whole-exome sequencing to clone deafness genes, cell type–specific expression patterns can provide valuable information, specifically when multiple changes are seen within a single linkage interval.

### Identification of miRNAs that regulate the inner ear transcriptome

Expression of miRNAs is essential for the development of the inner ear. Specifically, mutations in miR-96 have recently been found to underlie hereditary non-syndromic hearing loss in humans and mice [11]–[13]. miRNAs modulate the expression of genes by affecting either the translation of mRNA to protein or the stability of mRNAs. Previous studies demonstrate that activity of miRNAs in specific biological conditions can be inferred from comparative analysis of mRNA expression profiles [14], [15]. The premise of this *in silico* approach is that each miRNA typically down-regulates the expression of dozens of target mRNAs, thereby leaving a molecular signature on the cellular transcriptome that marks its activity. Significant down-regulation of miRNA targets in a certain cell population therefore implies enhanced activity of that miRNA in the biological condition of interest.

**Figure 4:**
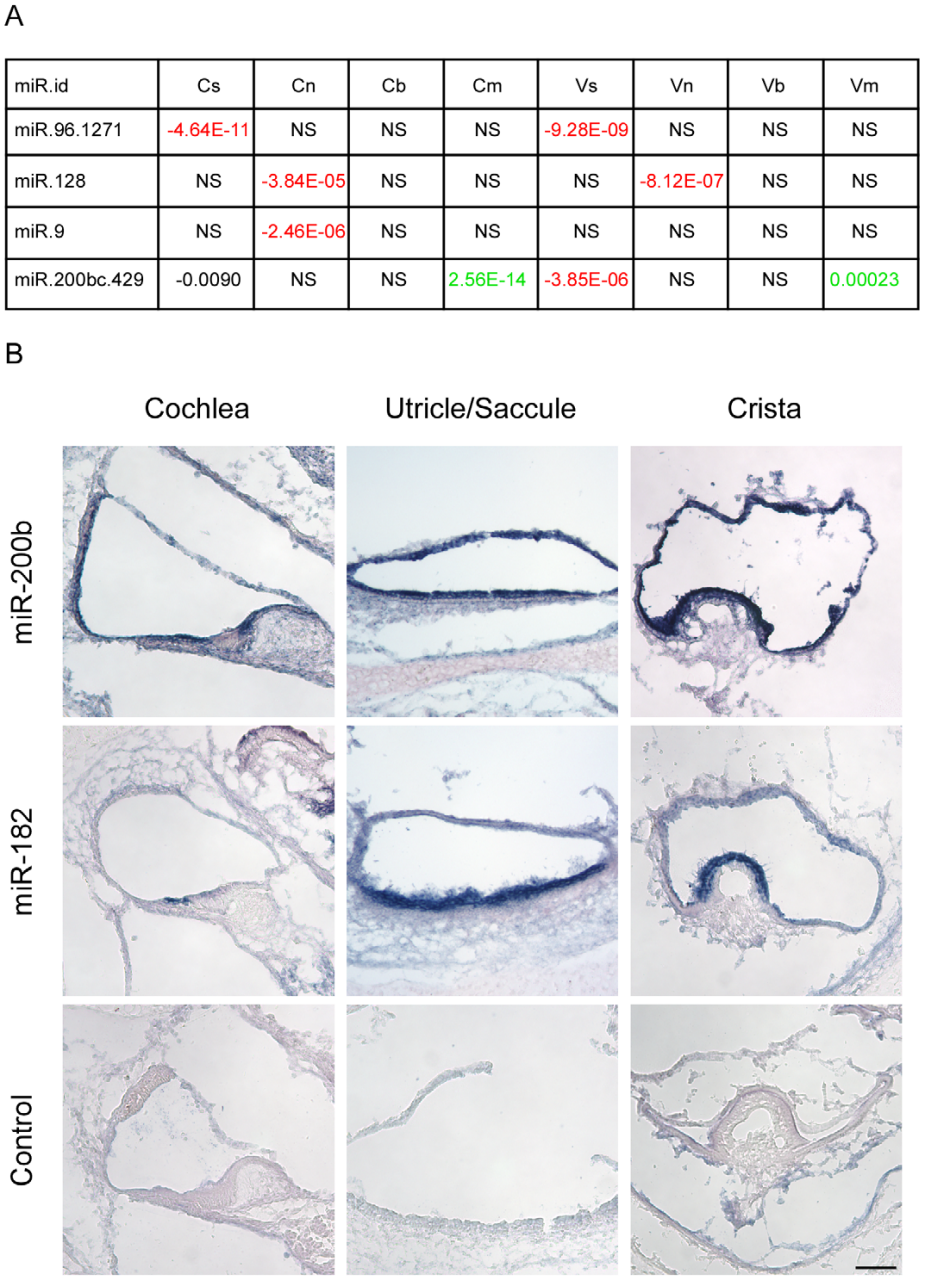
Identification of cell type–specific miRNA in the newborn mouse inner ear. [A] miRNAs whose predicted targets were significantly down-regulated in specific cell types. Each cell reports p-value for the comparison in the indicated cell-type between fold-change distribution of the targets of the indicated miRNA and all the rest of genes (see Materials and Methods). Negative sign indicates down-regulation of the miRNA targets; positive – up-regulation; NS = Not significant difference (p-value>10-5). [B] miR200b is expressed in all epithelial cells of the newborn mouse inner ear. Sections of whole mount in situ hybridizations that were performed on newborn mouse inner ears, with probes for miR200b, miR182 (as a hair cell-specific positive control) and no-probe control. Inner ears were then sectioned. Representative sections from the cochlear duct, otolith organs and crista ampullaris are shown. Scale bar  =  150 µm.

Our *in silico* search for miRNAs detected four candidate families whose predicted targets were depleted in a statistically significant manner in selected cell populations (Figure 4A). Reassuringly, the top-scoring miRNA identified by this analysis was miR-96. In comparison with other genes, the set of predicted targets of miR-96 was significantly down-regulated in sensory epithelial cells both in the auditory and vestibular systems (p-value 4.64E-11 and 9.28E-09, respectively; Wilcoxon test). This bioinformatics analysis pinpointed miR-96 as a major regulator of gene expression in sensory cells of the inner ear, without using any prior knowledge of this organ, and provided a comprehensive list of putative targets of miR-96 in inner ear sensory epithelia (Table S6).

We also found that predicted targets of miR-128 were significantly down-regulated in the neurons of the auditory and vestibular systems, while the predicted targets of miR-9 were significantly down-regulated only in the neurons of the auditory system. Both miR-128 and miR-9 are known to have an important function in neuronal development [16].

Finally, the set of predicted targets of the miR-200b family were significantly down-regulated in sensory cells of the auditory and vestibular systems while being significantly up-regulated in mesenchymal cells. This finding strongly suggests that miR-200b is expressed and active in sensory epithelial cells while its levels are very low in mesenchymal cells of the inner ear, consistent with the documented role of the family of miR-200b in establishing and maintaining an epithelial phenotype of cells. To validate this result, whole mount *in-situ* hybridizations were performed on newborn inner ears of wild type mice with a probe for miR-200b, as well as a probe for miR-182 (a miRNA that is expressed in hair cells and ganglion cells of the ear [17]) as a positive control, and a no-probe negative control. As predicted by the *in silico* analysis, miR-200b is expressed in all of the sensory epithelial cells of the newborn mouse inner ear, both in the auditory and the vestibular systems (Figure 4B). Furthermore, the expression of miR-200b is epithelial-specific and included both the sensory and non-sensory epithelial cells.

### Identification of transcription factors that regulate the inner ear transcriptome

**Figure 5:**
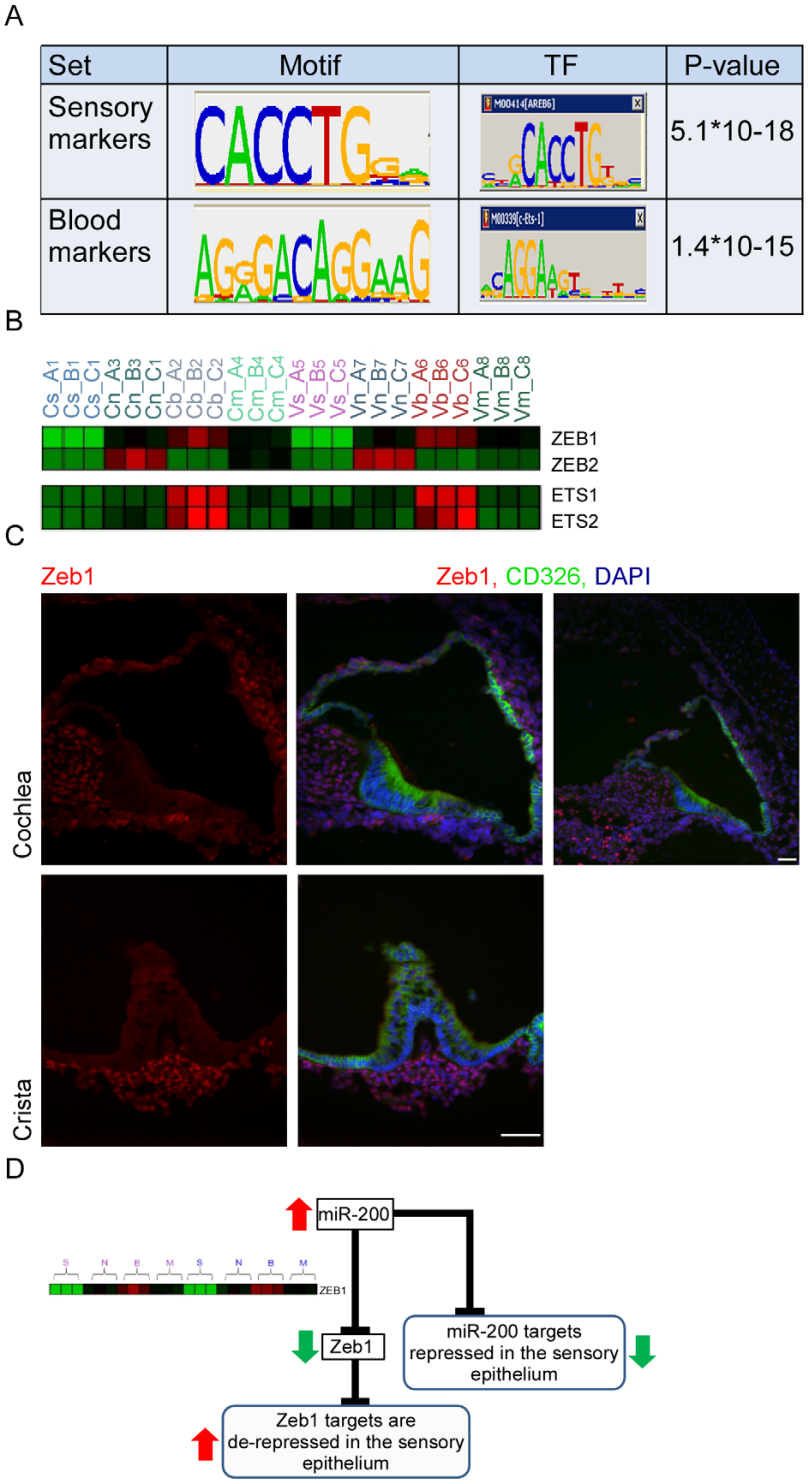
Key regulators of the inner ear transcriptome. [A] Enriched cis-regulatory motifs found in the promoters of marker genes. The motif enriched in the promoters of the sensory markers corresponds to the binding signature of Zeb1/2 transcription factors, while the motif enriched in the promoters of the endothelial markers corresponds to the binding signature of Ets1/2 transcription factors. “0” indicates no-enrichment. [B] Expression profiles of Zeb1/2 and Ets1/2 in our dataset are in full accord with the prediction of the motif enrichment analysis: Ets1/2 are highly expressed in endothelial cells while expression of Zeb1/2 is excluded from sensory cells. Color legend: red and green indicates increase and decrease in expression, respectively. [C] Zeb1 is expressed in the non-epithelial cells of the mouse inner ear. Sections of inner ears from newborn wild-type mice were stained with an antibody that detects Zeb1 (red), an antibody for CD326 (green) – which marks the epithelial cells in the mouse inner ear and DAPI to counter stain cell nuclei. Note that Zeb1 is not expressed in the epithelial cells of the inner ear (white asterisks). Upper right image – a low magnification image showing that Zeb1 is expressed in most of the non-epithelial cells, including the cells of the spiral ganglion (green asterisk). Scale bar  =  50 µm. See also Figure S5. [D] A model for the function of the miR-200 family in the sensory epithelium of the inner ear.

In order to identify transcription factors (TFs) that determine cell fate in the inner-ear, we applied a bioinformatics approach which, under the assumption that co-expression of transcripts over multiple conditions implies their transcriptional co-regulation, statistically searches for cis-regulatory elements that are over-represented in promoter regions of the sets of co-expressed genes [18]. We applied this approach to the sets of cell-type specific marker genes which were identified by our trasncriptomic analysis (see Materials and Methods, Figure S4 and Table S5). This *de novo* motif discovery analysis identified a top scoring motif that was significantly enriched in the set of promoters of the sensory marker genes (Figure 5A), and corresponds to the binding signature of the ZEB1 and ZEB2 transcription factors [19]. ZEB1 and ZEB2 are primarily repressors of transcription [20]. Thus, the enrichment of the ZEB1/2 binding signature in the promoters of sensory marker genes suggests that Zeb1 and Zeb2 are expressed in all cell types except sensory epithelial cells where their targets can be expressed due to lack of suppression. The second statistically significant DNA motif in our dataset was identified in the promoters of genes specifically expressed in vascular endothelial cells and corresponds to the binding signature of the c-Ets-1/2 transcription factors (Figure 5A). c-Ets1 and c-Ets2 are transcriptional activators, and therefore the enrichment of their binding signature in promoters of blood vessel markers suggests that these factors are specifically expressed in blood vessel cells. Examination of the mRNA expression patterns of *Zeb1, Zeb2*, *c-Ets1* and *c-Ets2* in our dataset confirmed these predictions, showing a depletion of *Zeb1* and *Zeb2* mRNA from the sensory epithelial cells and an enrichment of *c-Ets1* and *c-Ets2* mRNA in the vascular endothelial cells (Figure 5B). These results corroborate the computationally-derived hypotheses implicating major roles for Zeb1/Zeb2 and c-Ets1/c-Ets2 in suppressing an epithelial and enhancing a vascular endothelial phenotype, respectively, in the inner ear.

To validate the expression of Zeb1, inner ear sections from newborn wild-type mice were stained with an antibody against Zeb1 [21], and epithelial cells were labeled with CD326. As expected, all non-epithelial cells in the auditory and vestibular organs express Zeb1 while none of the epithelial cells show any Zeb1 expression, consistent with our *in silico* predictions. No expression was detected in the sections stained with the pre-immune serum (Figure 5C and Figure S5). Thus, our computational analysis identified the miR-200b-ZEB1/2 pathway as a key regulator of epithelial and mesenchymal identities in the inner ear (Figure 5D). Importantly, a major mechanism by which miR-200b achieves this function is direct down-regulation of *Zeb1* and *Zeb2* [22]. Our *in situ* hybridization results for miR-200b and immunolocalization for Zeb1 confirm that Zeb1 and miR-200b are expressed in mutually exclusive cell populations in the ear.

### Misregulation of the Zeb-1 pathway in the inner-ear of Twirler mice

**Figure 6:**
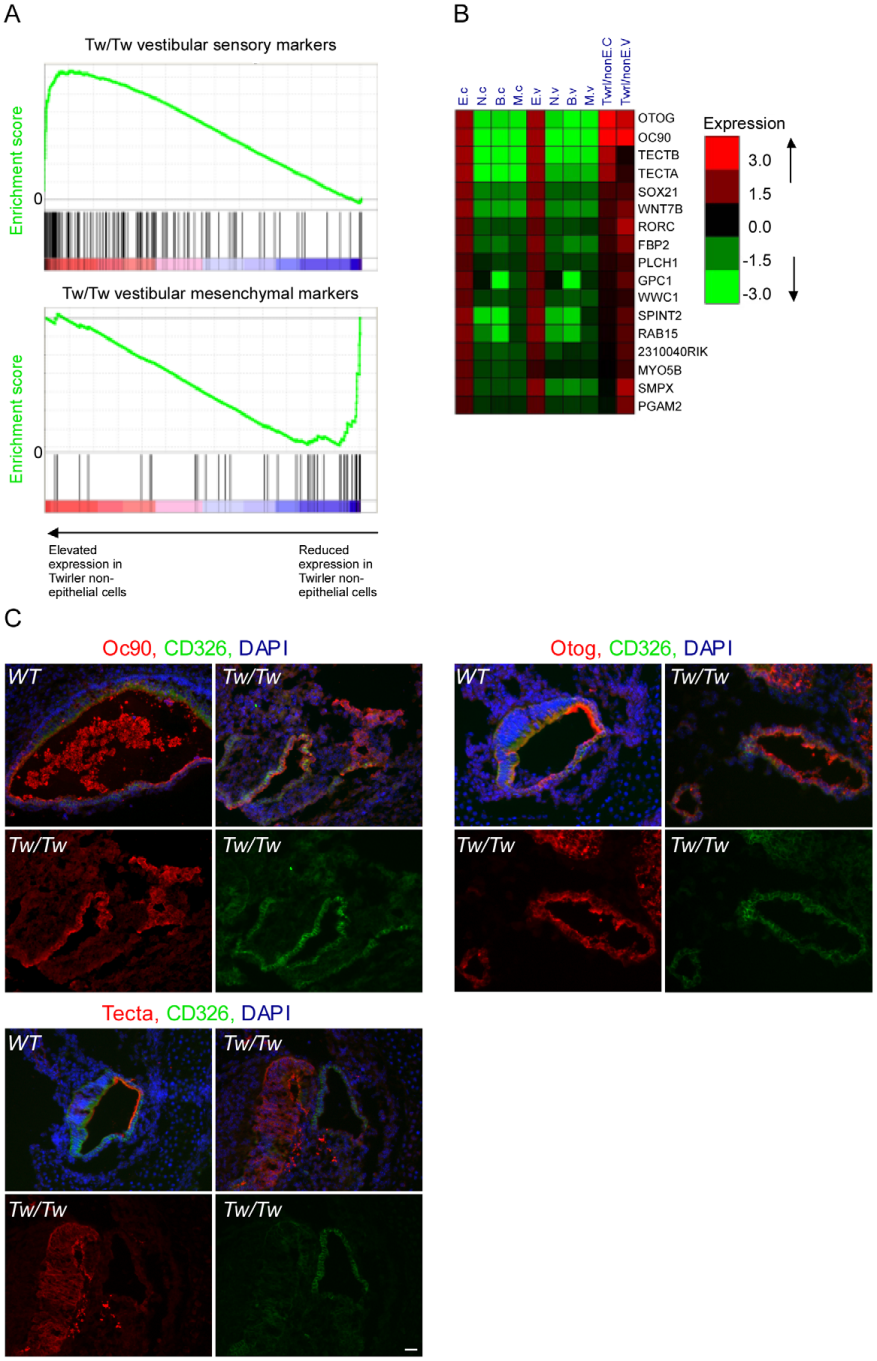
Deregulation of Zeb-1 pathway in the inner-ears of Twirler mutant mice. [A] Top panel – gene expression analysis of CD326-negative cells sorted from *Tw/Tw* mice show increase in epithelial specific markers in the *Tw/Tw* mutants compared with their wild type littermate controls. Genes were sorted along the X-axis according to their fold-change between the two genotypes, and the distribution of epithelial marker genes in this sorted list was examined using Gene-Set Enrichment Analysis (GSEA) tool [40] (location of the marker genes in the sorted list is indicated by vertical bars). The epithelial marker genes were significantly enriched among genes whose expression was elevated in *Tw/Tw* CD326-negative cells (p = 1.2*10^−15^, Wilcoxon test). Lower panel - gene expression analysis of CD326-negative cells sorted from *Tw/Tw* mice shows decrease in mesenchymal specific markers in the *Tw/Tw* mutants compared with their wild type littermate controls (p = 1.72*10^−10^). [B] Many putative Zeb1 targets are de-repressed in CD326-negative cells in *Tw/Tw* mice inner ear. Log2 fold of change of genes with differential expression in the CD326-negative cells of the *Tw/Tw* mice compared with their wild type littermate controls. All listed genes harbor a Zeb1 binding site within their promoters. [C] The expression of Oc90, otogelin and α-tectorin is altered in the *Tw/Tw* mice. In wild type mice Oc90, otogelin and α-tectorin are expressed in the extracellular matrices and epithelial compartment, here marked in green by CD326. In *Tw/Tw* mice a robust expression of Oc90, otogelin and α-tectorin is noted also in the non-epithelial compartment (areas with expression of Oc90, otogelin and α-tectorin which do not overlap with CD326 expression). In each of the three panels the upper two figures represent merged images of the staining of the altered gene, CD326 and DAPI in wild type (left) and *Tw/Tw* ears (right) and the lower two figures show the unmerged staining of the altered gene (left) and CD326 (right). Scale bar  =  50 µm.

Twirler mutant mice have, among other phenotypes, auditory and vestibular defects [23]. Recently, a single nucleotide change in the first intron of *Zeb1* was shown to underlie the phenotype of Twirler [24]. The mutation leads to an up-regulation of Zeb1 RNA and protein [24], although how this leads to the observed phenotype is still unresolved. We therefore hypothesized that if ZEB1 function is compromised in Twirler mice, many epithelial genes should be de-repressed and abnormally expressed in non-epithelial compartments. To test this hypothesis, auditory and vestibular epithelia were dissected from newborn *Tw/Tw* mice and their wild type littermate controls, dissociated and sorted into CD326-positive and -negative cells, which in wild type mice represent the epithelial and non-epithelial compartments. We compared the expression levels of the epithelial marker genes in CD326-negative cells of the *Tw/Tw* mice and their wild type controls. In full accordance with our model, the expression of epithelial markers was strikingly elevated in CD326-negative cells of the *Tw/Tw* mice (p = 3.7*10^−12^ for the cochlea and p = 1.29*10^−15^ for the vestibular system, Wilcoxon test), consistent with a severe misregulation of the ZEB1 pathway in Twirler inner ear (Figure 6A). Conversely, a relative down-regulation of mesenchymal marker transcripts was identified in the CD326-negative cells of the *Tw/Tw* mice.

We next examined the expression levels of epithelial markers in CD326-positive and negative cells dissected from the auditory and vestibular epithelia of *Tw/+* mice. Notably, while *Tw/+* mice have vestibular dysfunction associated with structurally abnormal semicircular canals, their hearing impairment is variable and the only observed auditory structural defect is shortening of the cochlear duct [24]. In accordance with the results we obtained for the *Tw/Tw* mice, the set of epithelial marker genes showed significantly elevated expression in the CD326-negative cells of the *Tw/+* vestibular systems compared with wild-type controls although the elevation was not as strong as it was in the *Tw/Tw* ears (p = 1.8*10^−7^ and p = 1.3*10^−15^, respectively, and Figure S6A). *Tw/+* cochlear ducts showed no significant change in expression of sensory markers, consistent with the incompletely penetrant and mild auditory phenotype in these mice (Figure S6B and [24]). Taken together, the cell type–specific transcriptomes of heterozygous and homozygous *Tw* auditory and vestibular organs support our hypothesis that the Tw mutation exerts its pathogenic affect via misregulation of the ZEB1 pathway.

Many of the epithelial marker genes that were misregulated in the *Tw/Tw* mice harbor a ZEB1 binding site signature in their promoters (Figure 6B). Interestingly, this group of genes contains several genes that encode for proteins that form the extracellular matrices of the mouse inner ear: *Tecta*, *Tectb*, *Otog*, and *Oc90* [25], [26]. Otogelin, and α- and β tectorins are necessary for proper formation of the tectorial membrane, a gelatinous matrix that covers that apical surface of the hair cells in the cochlea. This matrix contacts the hair cell stereocilia and is important for proper deflection of the stereocilia in response to sound. Otoconin-90 (Oc90) forms part of the organic component of the otoconia, small calcium carbonate-enriched protein matrices that are essential for sensing linear acceleration and gravity by the utricle and saccule, respectively. To validate these results, sections of inner ears from newborn Twirler mice and their littermate controls were stained with antibodies against α-tectorin, otogelin and Oc90. For all of these proteins, wild type expression was restricted to the epithelial compartment and the resulting extracellular matrices. In contrast, in Twirler mice, multiple cells expressing α-tectorin, Otogelin and Oc90 were found within the non-epithelial compartment, often in clusters (Figure 6C).

## Discussion

In this study we developed a protocol to isolate the major cell types of the auditory and vestibular organs of newborn wild type mouse inner ears. We applied this approach to characterize the tissue- and cell type–specific transcriptome of the newborn mouse inner ear, followed by a computational analysis to identify co-expressed genes and regulators of cell fate. The cis-regulatory motif analysis was carried out using a *de novo* motif discovery tool with no bias to any pre-selected TFs [18]. Searching all possible DNA motifs, the analysis defined only two statistically significant motifs, corresponding to ZEB1/2 (enriched in promoters of genes whose expression was reduced in sensory cells) and c-Ets-1/2 (enriched in promoters of genes whose expression was elevated in blood cells). Most of the reported successful analyses using similar approaches were achieved in lower organisms (primarily yeast) in which transcriptional regulation is much simpler [27], [28]. Additional regulators are likely to be identified by sorting the cell types we studied to even more homogeneous subpopulations (e.g. separating hair cells from supporting cells).

**Figure 7:**
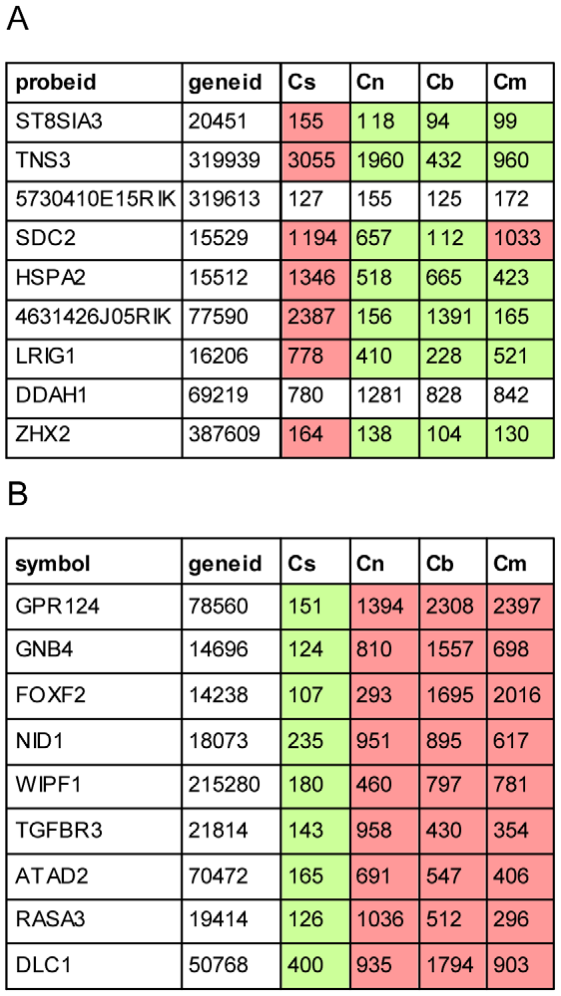
Cell type–specific targets of miR-96. Cell type–specific expression of the nine most likely miR-96 targets suggested by Lewis et al. (A) and according to our dataset (B). Cs, Cn, Cb and Cm represent expression values in sensory epithelial, neuronal, vascular endothelial and mesenchymal cells from newborn wild type cochleae. Green and red cells indicate relative decreased and increased expression relative to the average expression across all cell types, respectively.

The strength of this approach is further demonstrated by the ability to detect not only compartment specific regulators of cell fate (Zeb1/2, c-Ets1/2, miR-128, miR-9 and miR200b) but also miR-96, a miRNA which is expressed only in a subset of the sensory epithelial cells. A recent study by Lewis et al. identified putative targets of miR-96 by comparing expression profiles of whole auditory sensory tissues dissected from wild type and miR-96 mutant mice [12]. Interestingly, the miR-96 putative targets identified by Lewis et al. and by our study represent two non-overlapping groups. Further analysis of the cell type–specific expression patterns of these putative targets reveals that while most of the targets identified by Lewis et al. are elevated and co-expressed with miR-96 in the same cell population (sensory epithelium), our analysis identified targets whose expression is reduced in the sensory epithelium (Figure 7). This is consistent with the two functional effects of repression of targets by miRNAs: repression of leaky transcription, and buffering of transcriptional noise [29]. In the first case, the miRNA and its targets are expressed in mutually exclusive cell types. In the second case, the miRNA functions to reduce fluctuation in the expression of its target due to transcriptional noise, and therefore it is co-expressed with its targets in the same cells. Since miR-96 is expressed in the inner-ear specifically in hair cells and as the hair cells consist of only a small fraction of the total number of cells in this organ, only targets that are hair cell-specific or hair cell-enriched (and hence targets that belong to the second group) are likely to be detected when extracting RNA from intact sensory tissues (for example, tissues that consists of a mix of epithelial and non-epithelial cells, or a mix of hair cells and supporting cells) as was done by Lewis et al. For the other group of targets, the effect of miR-96 is expected to be diluted by their expression in the other inner-ear compartments. On the other hand, in our study, using a cell type–specific analysis of wild-type inner ears, we could only identify targets of miR-96 that are expressed also outside the sensory epithelial cells (and hence targets that belong to the first group). We hypothesize that repeating the experiment described by Lewis et al. using a cell type–specific approach would likely identify both groups of complementary target genes.

We applied our method to analyze ears from Twirler mutant mice to determine if the observed inner ear malformations were associated with a disruption of wild-type cell type–specific gene expression profiles. The results revealed misregulation of epithelial and mesenchymal specific genes in the non-epithelial compartment. This finding is consistent with a misregulation of Zeb1-regulated gene expression in the Twirler mutant mice. Furthermore, a group of genes that encodes the extracellular matrices of the mouse inner ear – *Otog, Tecta, Tectb* and *Oc-90*- was upregulated in the non-epithelial cells of the homozygous Twirler inner ears and harbored a binding site for Zeb1 in their promoter, suggesting that they are direct targets of the Zeb1 transcription factor in the developing inner ear mesenchyme. Of note, immunohistochemical analysis of inner ears from *Zeb1*-null mice [30] and their heterozygous or wild type littermate controls showed very subtle, if any, structural abnormalities in the ears of the *Zeb1*-null mice (Data not shown). It is possible that *Zeb2* may compensate for the loss of *Zeb1* in *Zeb1*-null but not *Tw/Tw* inner ear mesenchyme. This is consistent with the observation that *Tw* is not a loss-of-function allele of *Zeb1* [24]. The expression profiles of Twirler ears suggest that a pathologic disruption of epithelial and mesenchymal cell identities underlies the inner ear malformations observed in the Twirler mouse mutant, consistent with misregulation of the ZEB1 pathway. This could arise from a loss of mesenchymal cell identity leading to mesenchymal-epithelial transition (MET), a loss of epithelial cell identity leading to epithelial-mesenchymal transition (EMT), or a combination of these mechanisms. The exact mechanism leading to the misregulation of the ZEB1 pathway is still unknown, but likely results from loss of binding of one or more Myb proteins to a binding site disrupted by the Twirler *Zeb1* mutation [24]. Therefore, further work is required in order to elucidate the exact mechanism of the Twirler mutation. A limitation of our approach is the utilization of CD326 to separate the epithelial from the non-epithelial compartment in the Twirler mutant mice, as CD326 itself could be regulated by Zeb1. Nevertheless, we were unable to identify other epithelial markers with a stable pattern of expression to separate epithelial and non-epithelial cells.

Ultimately, to prove direct regulation of gene expression by transcription factors, one has to demonstrate protein-DNA interactions. This is specifically challenging when working with tissues or small organs such as the inner ear sensory epithelia. Future development of techniques to perform large-scale whole transcriptome analysis of protein-DNA interactions using small numbers of cells will enable combining cell type–specific approaches with techniques such as chromatin immunoprecipitation-sequencing (ChIP-Seq) [31]. Finally, implementation of our approach to study other mouse models for hearing loss will likely shed light on the molecular mechanisms downstream and upstream of many of the deafness genes.

## Materials and Methods

### Animal study approvals

All procedures involving animals were carried out in accordance with the *National Institutes of Health Guide for the Care and Use of Laboratory Animals* and have been approved by the Animal Care Committee at the University of Maryland, Baltimore (protocols 0107005 and 1209008) or the joint Animal Care and Use Committee of the National Institute of Neurological Disorders and Stroke and the National Institute on Deafness and Other Communication Disorders.

### Mice

Wild type ICR mice were obtained as time-mated animals from Charles River Laboratories (Maryland).

### Immunofluorescence

Newborn mice were euthanized by decapitation. Whole inner ears were micro-dissected out of the middle cranial fossa following the contour of their cartilaginous capsule, in a 3-cm dish with cold PBS. The tissue was then fixed in 2–4% paraformaldehyde (PFA) in PBS for 4 hours or overnight at 4°C. Cryoprotection was performed in a sucrose gradient (5%, 10%, 15%, 20%, 25% in PBS), 30 minutes in each step. The tissue was then kept at 30% sucrose in PBS overnight at 4°C. The following day, tissue was embedded in optimal cutting temperature (O.C.T.) compound (Tissue-Tek) and positioned with the lateral aspect of the basal turn of the cochlea at a 90° angle to the surface of a small plastic receptacle. The tissue was then frozen at this position using dry ice and kept in a −80°C freezer until further use. Five-to 7-µm sections were obtained using a Leica CM1850 cryostat and placed on superfrost/Plus slides (Fisherbrand). Sections were kept at −80°C until further use.

For immunofluorescence the slides were brought to room temperature and a liquid-repellent marker pen (Daido Sangyo) was used to circle the sections. After a 30-min wash with PBS and 0.5% Tween-20, sections were blocked with 10% normal goat serum (NGS) for 20 min at room temperature. Primary antibody incubation was performed for one hour at room temperature or overnight at 4°C. Following three 5-min washes, sections were incubated for 45 minutes with a goat anti-rabbit polyclonal antibody conjugated either to Alexa Fluor 488 or 546 at room temperature, in the dark. Slides were then washed two times in PBS, cell nuclei were stained by 10-min incubation with DAPI 1 µg/ml (KPL) and mounted using ProLong Gold antifade reagent (Invitrogen). Sections were visualized using a Nikon Eclipse E600 upright microscope equipped for brightfield and fluorescence. Images were acquired using a SPOT Diagnostics image acquisition system. Hair cells were counterstained with a rabbit polyclonal antibody for Myosin VI (Proteus Biosciences) at 1∶1000 dilution. The following rat monoclonal antibodies were used to detect CD proteins: Alexa Fluor 488 anti-mouse CD326 (Ep-CAM) 1∶100, PE anti-mouse CD34 (1∶200) and FITC anti-human/mouse CD49f (1∶200) (BioLegend). The following antibodies were also used to detect their respective proteins: anti-Zeb1 rabbit polyclonal antibody (1∶1000) (Spoelstra et al., 2006), anti-OC90 (1∶200) [32], anti-Otog (1∶1000) [33] (gifts from Dr. Christine Petit) and anti-tectorin-α (1∶1000) [34] (a gift from Dr. Guy P. Richardson). Alexa fluor 488 and 546 goat anti-rabbit antibodies were used for secondary detection (1∶1000) (Molecular Probes) and Alexa fluor 546 phalloidin (1∶300) was used to stain actin.

### Cell sorting

Auditory and vestibular tissues with their underlying mesenchyme were harvested from P0-P1 mouse inner ears and collected in 2 cm plastic dishes. The auditory epithelium consisted of the organ of Corti as dissected for a standard explant culture [35]. The vestibular epithelia consisted of the saccule, utricle and three cristae ampullaris, with a varying amount of semicircular canal epithelium. The tissue was then incubated with 0.5 mg/ml thermolysin (Sigma) in 25 mM Hepes medium for 20 minutes in a 37°C/5%CO_2_ humidified tissue culture incubator, for partial digestion of the extracellular matrix. The thermolysin was then aspirated and the epithelia were incubated with Accutase enzyme cell detachment medium (eBioscience) for 3 min in a standard tissue culture incubator followed by mechanical disruption of the tissue using a 23G blunt ended needle connected to a 1ml syringe, and then by another cycle of a 3-min incubation and mechanical disruption. Cellular dissociation was confirmed by direct visualization using an inverted tissue culture microscope. The reaction was stopped by adding an equal volume of complete medium containing 10% heat-inactivated fetal calf serum, 1% Hepes and 1% Glutamax in DMEM (no antibiotics). Cells were passed through a 40-µm cell strainer (BD) and washed in PBS. Cells were counted and viability was assessed using Trypan blue exclusion (Sigma). Usually less than 2% of the cells stained positive for Trypan blue. The dissociated cells were then stained with CD326-APC (1∶2,000), CD49f-alexa488 (1∶100) and CD34-PE (1∶200) (Biolegend) diluted in FACS buffer (PBS, 5% fetal calf serum) for 30 min at room temperature in the dark followed by a wash in FACS buffer. The cells were then sorted using a Beckman Coulter MoFlo XDB flow cytometer and cell sorter or BD FACSARIA Cell Sorter (BD Biosciences). A small amount of cells from each population was routinely re-analyzed by performing a second pass through the sorter to evaluate the purity of the sorted cells. For the analysis of epithelial and non-epithelial gene expression in the Twirler mutant mice and their littermate controls, inner ears from newborn mice were dissected and dissociated as described. The cells were then stained with the antibody for CD326 in order to sort the cells into the epithelial and non-epithelial compartments. For cell sorting of auditory and vestibular epithelia of newborn *Tw/Tw*, *Tw/+* and *+/+* mice (six, two and six ears, respectively) were dissected as described above. Due to the structural changes in the cochlear epithelia of the homozygous mutant mice, the cochlear dissection for this set of experiments included the entire cochlear duct (including the scala vestibuli and scala tympani) to guarantee consistency in the dissections.

### RNA and extraction and real time RT-PCR

Total RNA was extracted using the RNeasy Plus Micro Kit (Qiagen). Real time RT-PCR for CD326, CD49f and CD34 was performed as previously described [4]. Experiments were performed as two biologically independent replicates, with each biological experiment consisting of total RNA that was extracted from cells collected from at least 20 ears. Results were normalized to the expression of each gene in the cochlear neuronal cells.

### Whole-genome expression profiling

Whole-genome mouse mRNA expression profiles were recorded using the Illumina Bead Array system and MouseRef-6 v2.0 Expression BeadChips. These arrays contain more than 45K probes which collectively interrogate all RefSeq annotated mouse genes (>26K genes) and ∼ 7K RefSeq predicted genes. Ten to 20 ng of total RNA were used as starting material. Total RNA was pre-amplified with the Ovation RNA Amplification System V2 (NuGEN). The A total of 1.5 µg of cDNA was then processed and labeled following the manufacturer's protocol. 1.5 µg of labeled cDNA were used for hybridization. Total RNA and cDNA were quantified using the Agilent 2100 Bioanalyzer (Agilent) and labeled cDNA was quantified using a NanoDrop (Thermo Scientific) prior to hybridization. Samples from the sorted populations of wild type mice were obtained and processed from three biologically independent experiments. The whole genome expression profiling of the Twirler mice was performed as a single repeat.

### Analysis of expression arrays data

Expression profiles were recorded from eight cellular populations (four cell-types: sensory epithelial, neuronal, vascular endothelial and mesenchymal, isolated from two inner ear organs: the cochlea and the vestibular system) in triplicates using Illumina Mouse WG6v2 BeadChip arrays. Expression levels were calculated using Illumina's Bead-Studio package. Probes not readily detected in the dataset were filtered out using detection p-values assigned by Bead-Studio to each measurement and requiring that each probe be detected (p-value<0.01) in at least two samples. This criterion left 16,983 probes for subsequent analyses. Arrays were then normalized using quantile normalization. Expression data were then analyzed using the EXPANDER package [36]. Hierarchical clustering of the samples contained all the 16,983 expressed probes in the dataset and used the average-linkage method.

### Identification of differentially expressed genes

Differentially expressed genes in the dataset were identified using two-way ANOVA analysis with a stringent p-value cutoff of 10-5. 3,167 probes were significantly affected by either factor (cell-type or organ) or showed significant interaction between these two factors (i.e. different cell-type expression patterns between the two organs).

### Clustering

Main expression patterns exhibited by the set of differentially expressed genes were detected by cluster analysis using the k-means algorithm. Prior to clustering, the expression level of each probe was standardized to mean = 0 and SD = 1. Enriched Gene Ontology (GO) categories in the clusters were sought using the TANGO algorithm implemented in EXPANDER.

### Definition of sets of marker genes

As clustering inherently generates a noisy division of probes and genes according to their expression patterns, we also defined more homogenous sets of cell-type specific markers. The set of marker genes of each cell-type included all the genes whose minimal expression level in the samples of that cell-type was at least 50% higher than its expression across all the rest of the samples. The expression patterns obtained by this division of genes were similar to those obtained by the clustering analysis (Figure S4 and Table S5).

### Identification of expression-interaction modules

Identification of sets of genes which both 1) share similar expression patterns across the cell types in our dataset and 2) are physically connected in the cellular protein-protein interaction web, was carried out using the MATISSE algorithm [10] which is implemented in the EXPANDER package.

### Identification of over-represented sequence motifs

Over-represented cis-regulatory sequence motifs in promoter regions (−1000 to +200 nt relative to transcription start site (TSS)) of the sets of cell-type marker genes were searched using AMADEUS [18]. The entire set of promoters of genes expressed in the dataset served as the background set in this analysis.

### Identification of active miRNAs

miRNA activity was statistically sought by comparing, for each miRNA family and in each cell-type, the relative expression levels between the set of predicted targets of the miRNA and the background set containing all the rest of genes [14]. Significant down-regulation of predicted targets of a particular miRNA in a particular cell population suggests that the activity of the miRNA itself is enhanced in those cells. Predicted miRNA targets were obtained from TargetScanS [37]. Prior to carrying out these tests, the expression level of each gene was normalized to its average level across the eight conditions in our dataset to obtain relative levels.

### Deafness-related loci

A list of cloned human deafness-related genes and deafness loci for which the underlying gene was not cloned yet was compiled from the Hereditary Hearing Loss Homepage (http://hereditaryhearingloss.org/). Human genes located within deafness loci were extracted using a Perl script. Mouse homologues of these genes were found using NCBI's HomoloGene [38].

### Identification of miR96 targets in the dataset of Lewis et al

Expression data were downloaded from Array-Express DB, quantile normalized and averaged over replicates. Expression ratios were then calculated between mean levels in the miR-96 mutant and wild-type samples.

### In situ hybridization

Whole-mount in-situ hybridization was performed with probes mmu-miR-182 and mmu-miR-200b (Exiqon) to detect miRNA-182 and 200b, respectively, as previously described [39]. No-probe experiments were performed as negative controls.

## Supporting Information

Figure S1 A novel cell type–specific protocol to sort the inner ear sensory organs. Related to Figure 1. [A] FACS plot analysis from newborn auditory and vestibular epithelia of wild type mice. Cells from the auditory and vestibular epithelia are sorted based on expression of CD326 and further divided based on the expression of CD49f and CD34. [B] Bar diagram summarizing the percent of cells contributing to each of the major cellular compartments in the auditory and vestibular epithelia. Values are an average of five biologically independent replicates. Error bars represent one standard deviation. One and two asterisks indicates p-values <0.05 and <0.01, respectively. [C] Semi-quantitative real time RT-PCR data testing for enrichment of CD326, CD39f and CD34 in the sensory (S), neuronal (N), vascular endothelium (BV) and mesenchymal (M) cells sorted from the cochlear (C) and vestibular (V) tissues. Expression data were normalized to the expression of each mRNA in the sensory epithelial cells of the cochlea.
(TIF)

Figure S2 Cell type–specific expression of candidate genes in the AUNA1 deafness locus. [A] The list of the RefSeq genes in the AUNA1 locus; Data were obtained from the UCSC Genome Browser on Human Feb. 2009 (GRCh37/hg19) Assembly. The locus was defined by D13S153 and D13S1317. [B] Mouse orthologs of the genes listed in [A] that are detected as expressed in the mouse inner ear based on our dataset. Of note, only two genes are selectively expressed in the neuronal cells consistent with a potential role in auditory neuropathy (marked in orange). One of these genes, *DIAP3*, was recently identified as the gene underlying this disorder [9].
(TIF)

Figure S3 MATISSE Modules. Expression-interaction modules identified in our dataset by the MATISSE algorithm. Related to Figure 3. Each module contains genes that are both 1) similarly expressed in our datasets and 2) physically connected in the cellular protein-protein web. In this figure, each module is represented by the mean expression pattern of the genes it includes (± SD).
(TIF)

Figure S4 Cluster analysis of marker genes. Related to Figure 5. Main expression patterns of marker genes as identified by k-means clustering. Each cluster is represented by its mean expression pattern ± SD. (Prior to clustering, gene expression levels were standardized to mean = 0, SD = 1. Y-axis in the clusters view shows the standardized levels). At the top of each pattern, the title indicates the cluster number and the number of probes assigned to the cluster.
(TIF)

Figure S5 Zeb1 is expressed in the non-epithelial cells of the mouse inner ear. Sections through the apical turn of a newborn cochlear duct were stained with an antibody that detects Zeb1 or the pre-immune serum (red), an antibody for CD326 (green) – which marks the epithelial cells in the mouse inner ear, and DAPI to counter stain cell nuclei. While Zeb1 was detected in the non-epithelial cells when sections were stained with the Zeb1 antibody, no Zeb1 expression could be detected when the sections were stained with the pre-immune serum. See also Figure 4.
(TIF)

Figure S6 Expression of epithelial markers in CD326-negative auditory and vestibular cells of *Tw/Tw* and *Tw*/+ mice. Changes in expression levels of sensory marker genes in the CD326-negative cells of the vestibular [A] and auditory [B] systems of *Tw/Tw* and *Tw/+* mice compared with the change in expression of the other genes in the same cell type (right side of each graph). The epithelial marker genes were defined by our cell-type transcriptomic analysis of wild type inner-ear. The background sets contained all the other genes which were detected as expressed in the dataset, but are not defined as epithelial marker genes. In the vestibular system, both in *Tw/Tw* and in *Tw/+* the expression level of epithelial marker genes is significantly elevated in CD326-negative cells, compared with the rest of the genes [A]. In the auditory system, the set of epithelial markers show a significant elevation in the *Tw/Tw* but not in the *Tw/+* mice [B].
(TIF)

Table S1 Differentially expressed genes in the newborn mouse auditory and vestibular sensory epithelia.
(XLSX)

Table S2 Cluster analysis of all differentially expressed genes.
(XLSX)

Table S3 Deafness genes detected as expressed in the dataset.
(XLSX)

Table S4 Cell type–specific mRNA expression levels of genes that map to uncloned deafness loci.
(XLSX)

Table S5 Cell type–specific markers.
(XLSX)

Table S6 List of miR-96 putative targets.
(XLSX)
