# Supplementary figures and images for: Cell Type–Specific Transcriptome Analysis Reveals a Major Role for Zeb1 and miR-200b in Mouse Inner Ear Morphogenesis
Source: PLoS Genet. 2011 Sep 29;7(9):e1002309. doi: 10.1371/journal.pgen.1002309 (PMC3183091; doi:10.1371/journal.pgen.1002309)

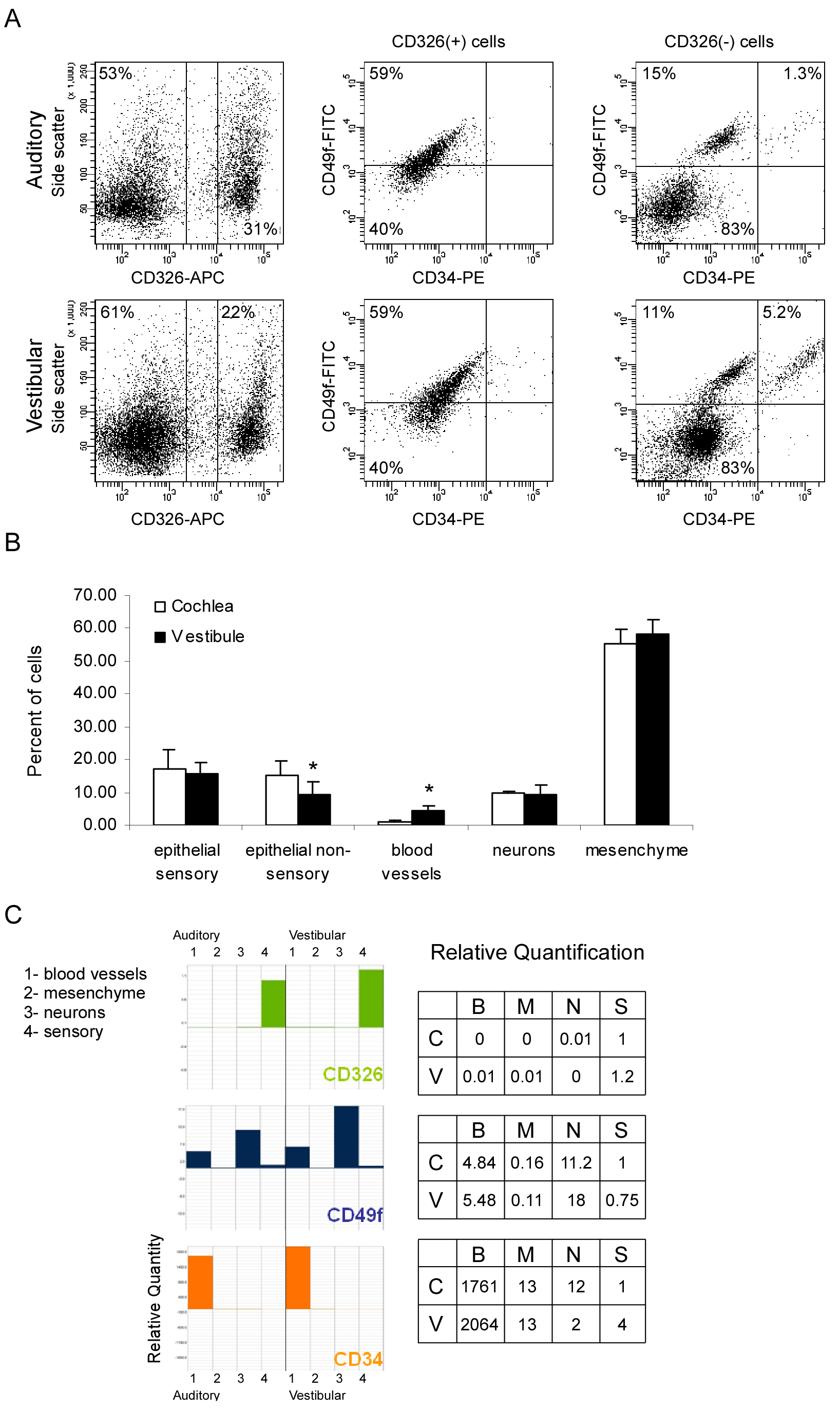

Supplement: Figure S1 — A novel cell type–specific protocol to sort the inner ear sensory organs. Related to Figure 1. [A] FACS plot analysis from newborn auditory and vestibular epithelia of wild type mice. Cells from the auditory and vestibular epithelia are sorted based on expression of CD326 and further divided based on the expression of CD49f and CD34. [B] Bar diagram summarizing the percent of cells contributing to each of the major cellular compartments in the auditory and vestibular epithelia. Values are an average of five biologically independent replicates. Error bars represent one standard deviation. One and two asterisks indicates p-values <0.05 and <0.01, respectively. [C] Semi-quantitative real time RT-PCR data testing for enrichment of CD326, CD39f and CD34 in the sensory (S), neuronal (N), vascular endothelium (BV) and mesenchymal (M) cells sorted from the cochlear (C) and vestibular (V) tissues. Expression data were normalized to the expression of each mRNA in the sensory epithelial cells of the cochlea. (TIF) [file pgen.1002309.s001.tif]

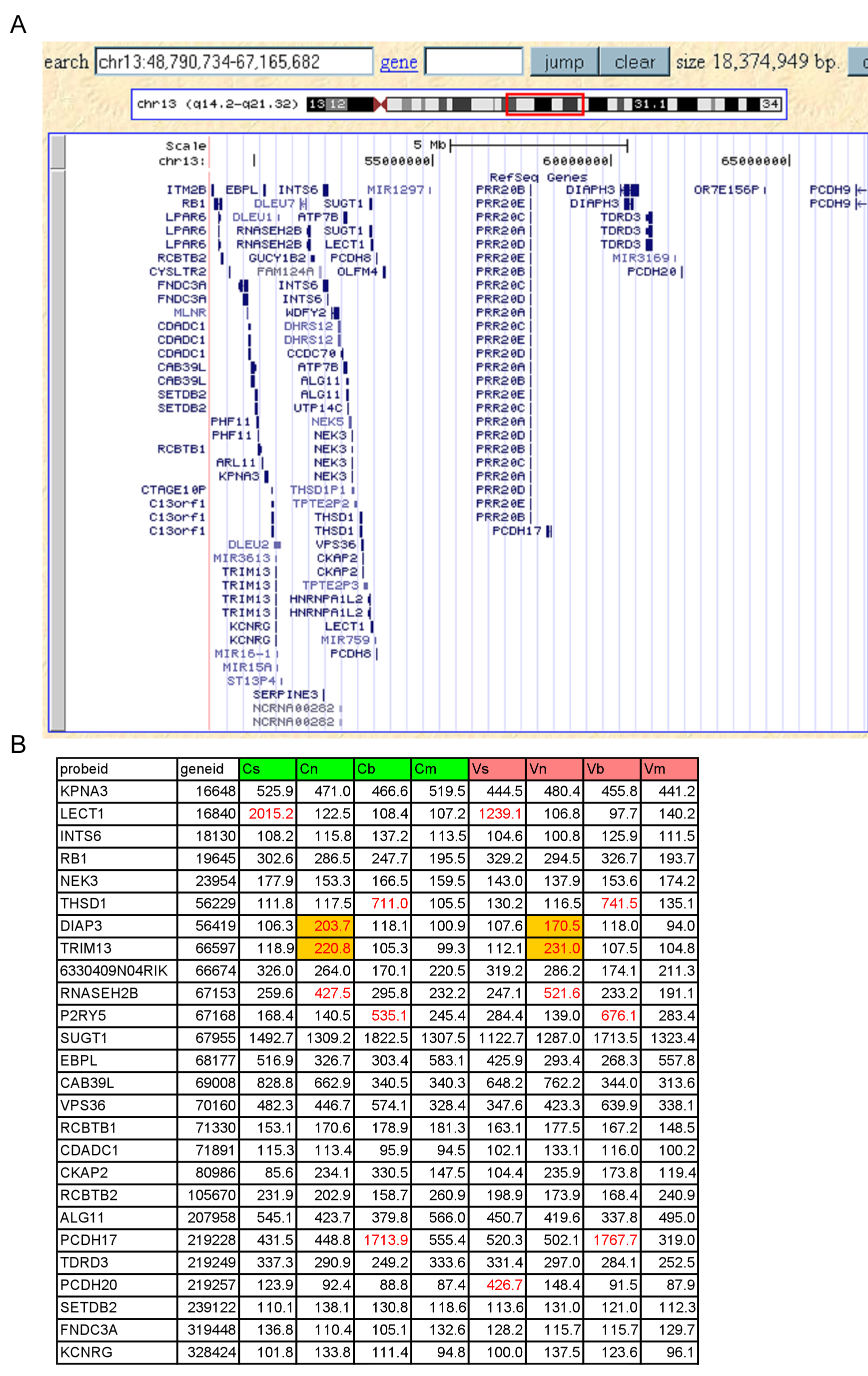

Supplement: Figure S2 — Cell type–specific expression of candidate genes in the AUNA1 deafness locus. [A] The list of the RefSeq genes in the AUNA1 locus; Data were obtained from the UCSC Genome Browser on Human Feb. 2009 (GRCh37/hg19) Assembly. The locus was defined by D13S153 and D13S1317. [B] Mouse orthologs of the genes listed in [A] that are detected as expressed in the mouse inner ear based on our dataset. Of note, only two genes are selectively expressed in the neuronal cells consistent with a potential role in auditory neuropathy (marked in orange). One of these genes, DIAP3, was recently identified as the gene underlying this disorder [9]. (TIF) [file pgen.1002309.s002.tif]

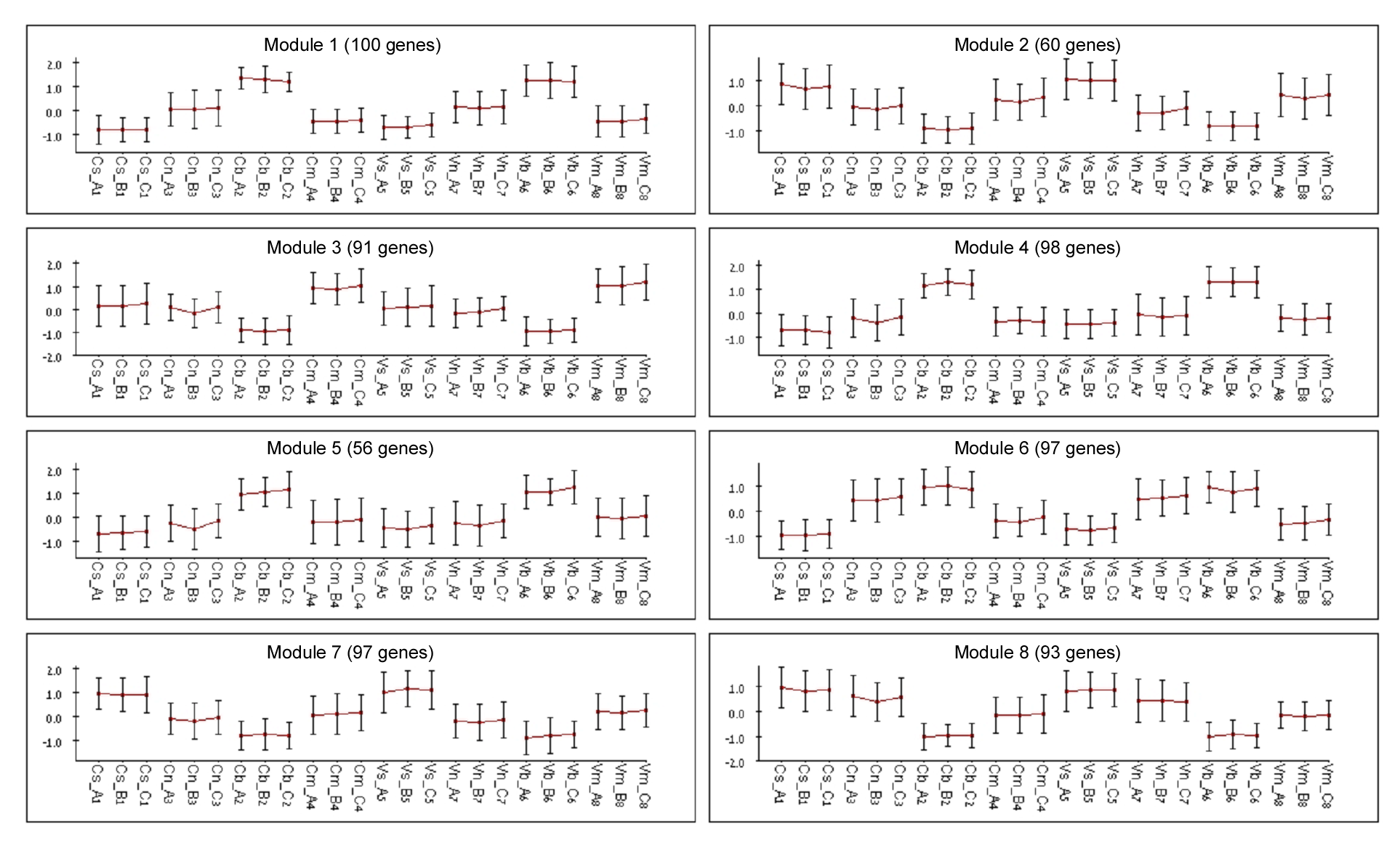

Supplement: Figure S3 — MATISSE Modules. Expression-interaction modules identified in our dataset by the MATISSE algorithm. Related to Figure 3. Each module contains genes that are both 1) similarly expressed in our datasets and 2) physically connected in the cellular protein-protein web. In this figure, each module is represented by the mean expression pattern of the genes it includes (± SD). (TIF) [file pgen.1002309.s003.tif]

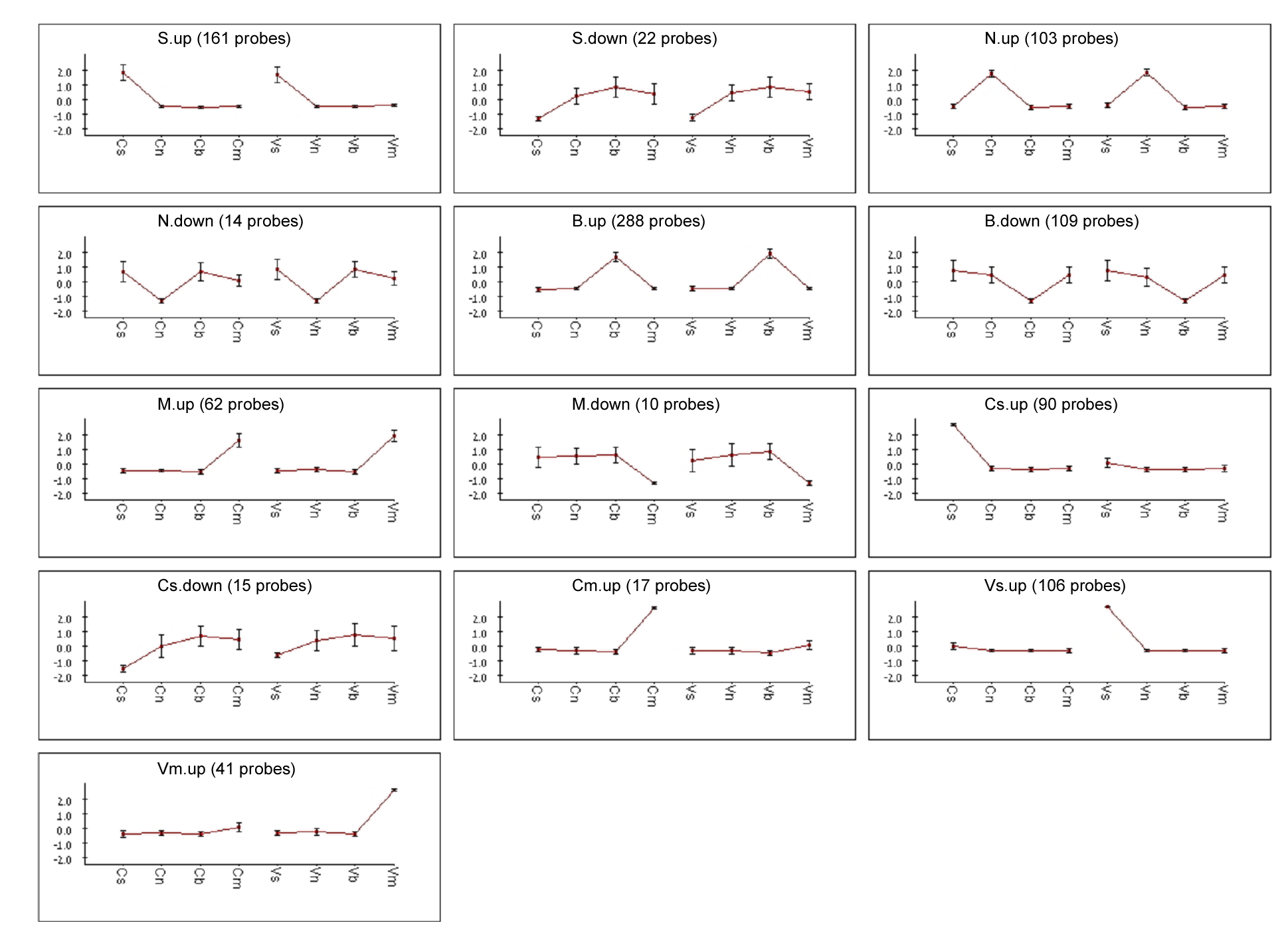

Supplement: Figure S4 — Cluster analysis of marker genes. Related to Figure 5. Main expression patterns of marker genes as identified by k-means clustering. Each cluster is represented by its mean expression pattern ± SD. (Prior to clustering, gene expression levels were standardized to mean = 0, SD = 1. Y-axis in the clusters view shows the standardized levels). At the top of each pattern, the title indicates the cluster number and the number of probes assigned to the cluster. (TIF) [file pgen.1002309.s004.tif]

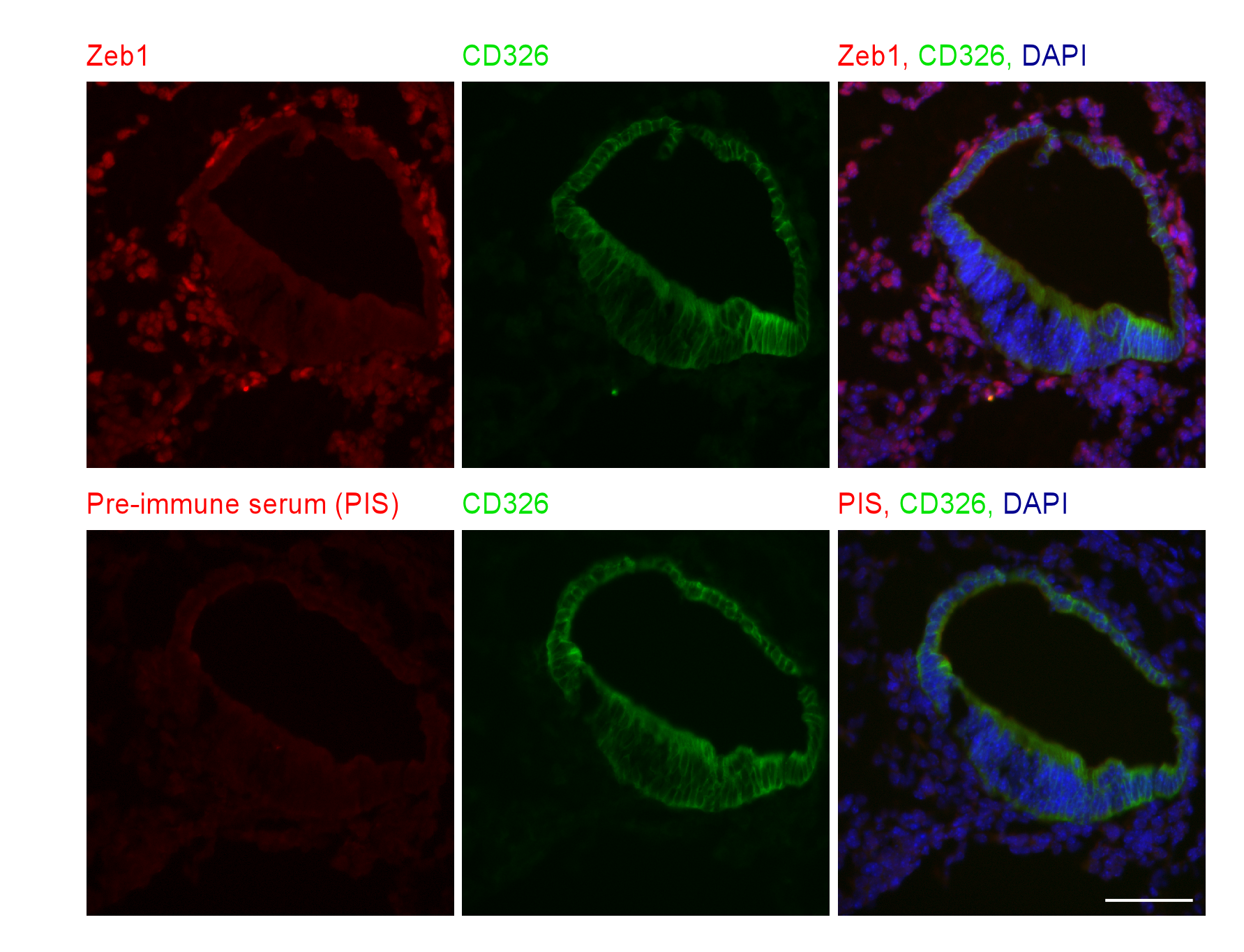

Supplement: Figure S5 — Zeb1 is expressed in the non-epithelial cells of the mouse inner ear. Sections through the apical turn of a newborn cochlear duct were stained with an antibody that detects Zeb1 or the pre-immune serum (red), an antibody for CD326 (green) – which marks the epithelial cells in the mouse inner ear, and DAPI to counter stain cell nuclei. While Zeb1 was detected in the non-epithelial cells when sections were stained with the Zeb1 antibody, no Zeb1 expression could be detected when the sections were stained with the pre-immune serum. See also Figure 4. (TIF) [file pgen.1002309.s005.tif]

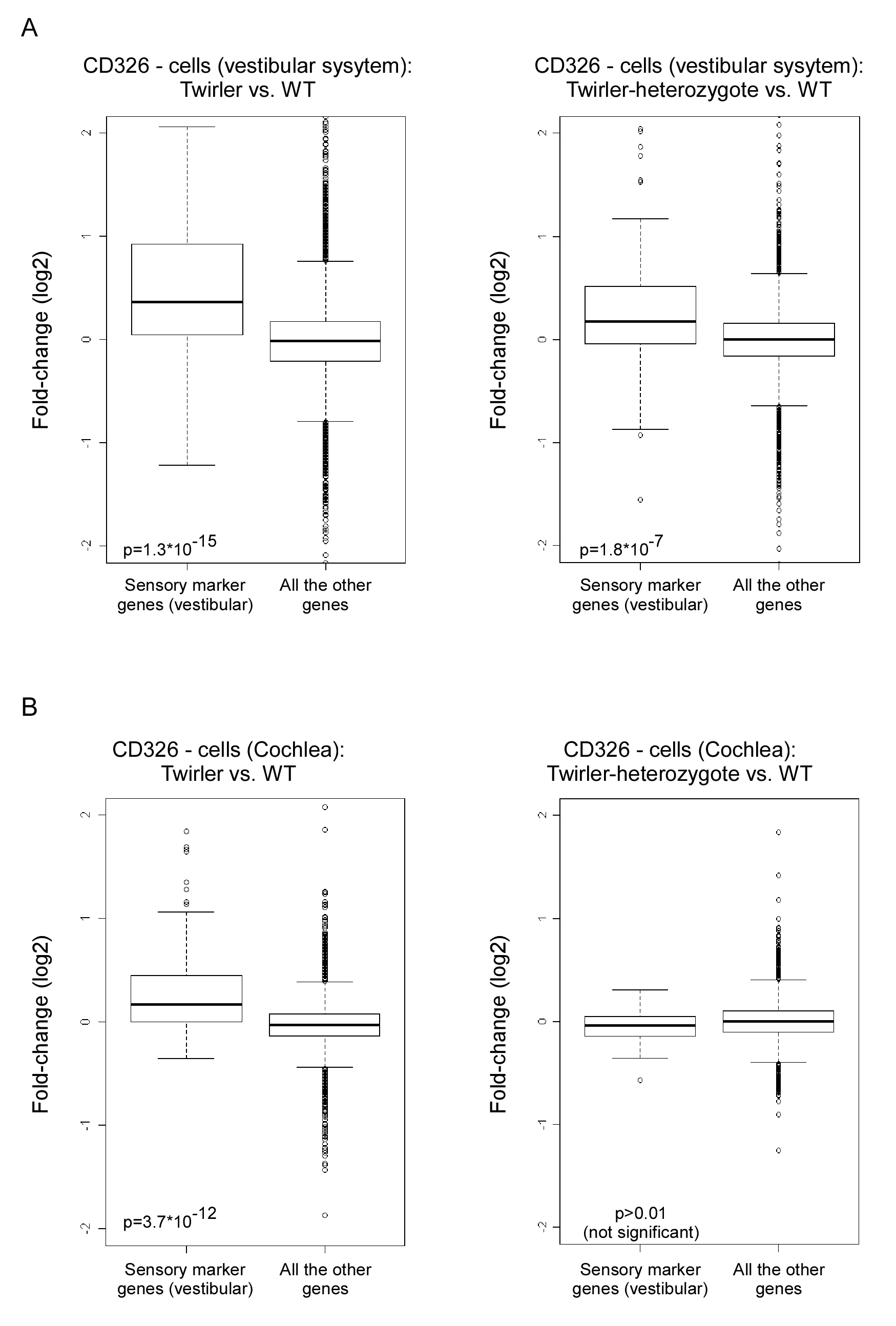

Supplement: Figure S6 — Expression of epithelial markers in CD326-negative auditory and vestibular cells of Tw/Tw and Tw/+ mice. Changes in expression levels of sensory marker genes in the CD326-negative cells of the vestibular [A] and auditory [B] systems of Tw/Tw and Tw/+ mice compared with the change in expression of the other genes in the same cell type (right side of each graph). The epithelial marker genes were defined by our cell-type transcriptomic analysis of wild type inner-ear. The background sets contained all the other genes which were detected as expressed in the dataset, but are not defined as epithelial marker genes. In the vestibular system, both in Tw/Tw and in Tw/+ the expression level of epithelial marker genes is significantly elevated in CD326-negative cells, compared with the rest of the genes [A]. In the auditory system, the set of epithelial markers show a significant elevation in the Tw/Tw but not in the Tw/+ mice [B]. (TIF) [file pgen.1002309.s006.tif]
